# Guanfacine poisoning resulting in transient ST-segment elevation: a case report

**DOI:** 10.1186/s12245-024-00634-0

**Published:** 2024-04-26

**Authors:** Ichiro Hirayama, Yoshito Kamijo, Hiroko Abe, Minaho Nonaka, Tetsuhiro Yano, Mitsuru Ishii, Yoshiteru Tominaga

**Affiliations:** 1 Department of Clinical Toxicology, Faculty of Medicine, Saitama Medical University, Saitama, Japan; 2 Department of Emergency Medicine, National Hospital Organization Saitama Hospital, 2-1 Suwa, Wako, Saitama, 351-0102 Japan; 3 Biodesign Inc, Tokyo, Japan

**Keywords:** Attention-deficit hyperactivity disorder, Guanfacine, Poisoning, ST-segment elevation

## Abstract

**Background:**

Guanfacine is an alpha-2 adrenergic agonist that decreases norepinephrine release and sympathetic outflow. With the increased use of guanfacine for attention-deficit hyperactivity disorder (ADHD), reports of guanfacine poisoning have also risen.

**Case presentation:**

A 15-year-old male (height: 170 cm, weight: 48 kg), who was taking 2 mg/day of guanfacine for ADHD, was brought to our emergency department after ingesting 40 tablets of guanfacine due to poor exam results. He presented with impaired consciousness and sinus bradycardia on an electrocardiogram (ECG), leading to diagnosis of guanfacine poisoning. Gastric lavage (5 L) was performed, and activated charcoal was administered. Although his consciousness gradually recovered, he developed ST-segment elevation on the ECG. Despite the absence of chest pain and elevated myocardial enzymes, coronary artery stenosis was not observed on coronary artery computed tomography. As his blood guanfacine level decreased, his ECG returned to normal.

**Conclusions:**

This case highlights the need for careful monitoring of guanfacine poisoning patients due to the potential for various cardiovascular events.

## Background

Guanfacine is an alpha-2 adrenergic agonist that reduces norepinephrine release and sympathetic outflow [1]. With its increased use for attention-deficit hyperactivity disorder (ADHD), reports of guanfacine poisoning have also risen [2]. Symptoms of guanfacine poisoning include somnolence, bradycardia, and hypotension due to its sympatholytic effects [1]. Guanfacine poisoning is associated with cardiac events, as prolonged QTc has been reported on electrocardiograms (ECG) of patients with guanfacine poisoning [3].

Here, we present a case of a 15-year-old male with guanfacine poisoning, exhibiting transient ST-segment elevation in the ECG.

## Case presentation

A 15-year-old male (height: 170 cm, weight: 48 kg) who was taking guanfacine 2 mg/day owing to suffering from ADHD was brought to our emergency department. He impulsively took 40 tablets of guanfacine because of his poor exam results at school. His vital signs on arrival were blood pressure 120/87 mmHg, pulse 42 beats per minute, regular respiration rate 24 breaths per minute, blood oxygen saturation 99% without oxygenation, and body temperature 36.8 °C. His Glasgow Coma Scale score was eye opening 2, verbal response 5, and motor response 6. ECG showed sinus bradycardia. As a result, he was diagnosed with guanfacine poisoning. Computed tomography (CT) revealed a drug lump in his stomach; gastric lavage of 5 L was therefore performed and activated charcoal was administered. The patient was then admitted to the intensive care unit for monitoring for state of consciousness and bradycardia.

**Fig. 1:**
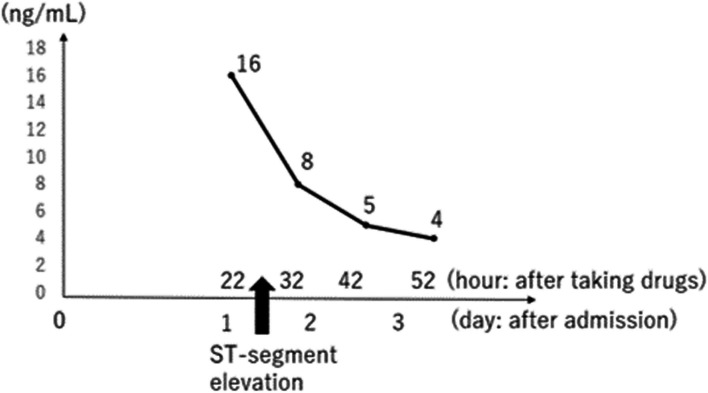
Time course of blood guanfacine concentration. ST-segment elevation occurred 28 h after taking 40 tablets of guanfacine

**Fig. 2:**
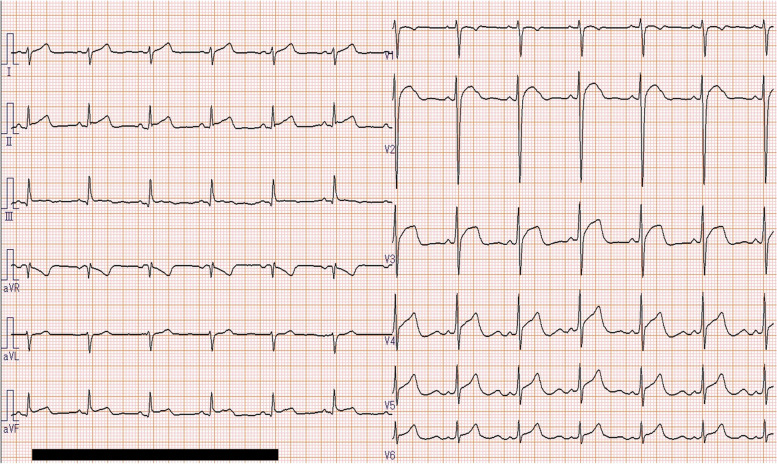
Patient electrocardiogram on hospital day 2 (ST-segment elevation observed from V2 to V6)

**Fig. 3:**
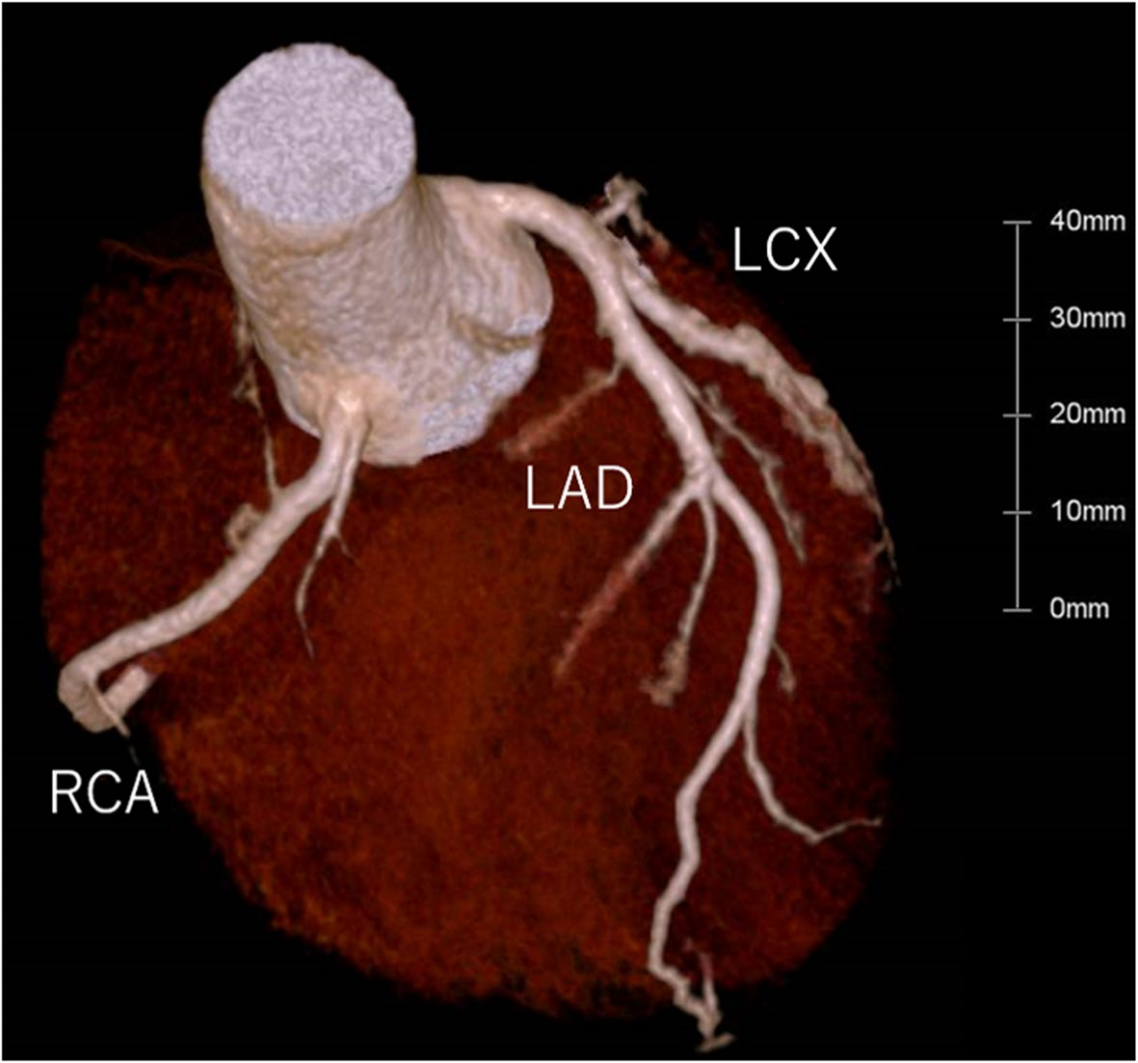
Coronary artery computed tomography. RCA, right coronary artery; LAD, left anterior descending artery; LCX, left circumflex coronary artery. There were no significant stenotic lesions observed in the coronary arteries

**Fig. 4:**
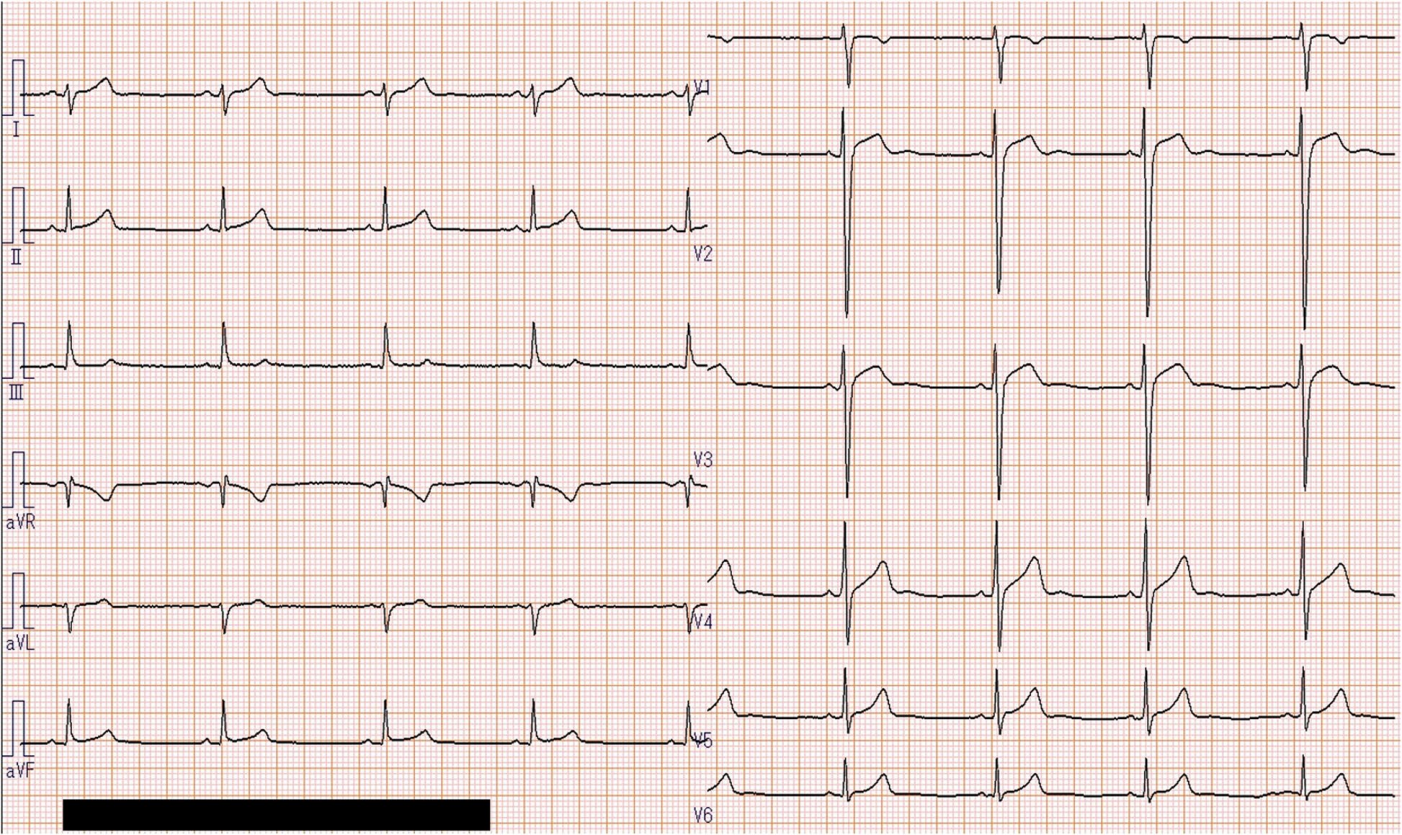
Patient electrocardiogram on hospital day 3, recovered to normal

The patient recovered consciousness as the blood concentration of guanfacine decreased (Fig. 1); however, ST-segment elevation occurred on an ECG on the 2nd hospital day (28 h after overdosing on guanfacine) as shown in Fig. 2. He had no chest pain and no elevation of myocardial enzymes. Coronary artery stenosis was not observed on coronary artery CT (Fig. 3), and he was kept under observation. ECG became normal on the 3rd hospital day (Fig. 4). Oral administration of guanfacine 2 mg was resumed on the 5th hospital day. He was discharged on the 8^th^ hospital day after his vital signs were considered stable.

## Discussion

We presented a case of a guanfacine poisoning patient who experienced transient ST-segment elevation during hospitalization. Guanfacine, an alpha-2 agonist, not only causes hypotension and bradycardia due to the interruption of sympathetic outflow, but it has also been revealed to occasionally cause a transient decrease in ventricular wall motion. Therefore, patients with guanfacine poisoning should be carefully monitored for changes in their ECG after admission.

Guanfacine received FDA approval for the treatment of ADHD in 2009, leading to its significant increase in availability and exposure to the public [2]. It has a half-life of 10 to 30 h, which varies based on kidney and liver functions [1]. Guanfacine is affected by drug interactions with CYP3A4 inhibitors or inducers [4]. Guanfacine poisoning is frequently associated with cardiac events. Common cardiovascular symptoms of guanfacine poisoning include hypotension, bradycardia, and prolonged QTc [5]. In addition, previous reports have shown that hypotension and bradycardia requiring vasopressors or atropine were rare, but there have been cases of cardiogenic pulmonary edema requiring intubation [5, 6].

The novelty of this case lies in the occurrence of transient ST-segment elevation in the anterior precordial lead on the ECG. Previous literature has demonstrated that alpha-2 adrenoceptor stimulation can lead to a transient decrease in anterior wall motion related to an epinephrine surge in rats exposed to psychological stress [7]. Although the patient’s blood concentration of guanfacine decreased over time, it remained significantly higher compared to that in adults taking the normal dose of guanfacine [8, 9]. When the patient’s blood guanfacine concentration approached the range of normal guanfacine dosage, the ECG changes resolved. This case is a rare instance of guanfacine poisoning where the transient ST-segment elevation, indicative of a decrease in anterior wall motion, was also observed in humans.

In conclusion, guanfacine poisoning patients should be carefully monitored after admission for its potential of causing a variety of cardiovascular events.

## Data Availability

No datasets were generated or analysed during the current study.

## Abbreviations

ADHD: Attention-deficit hyperactivity disorder
ECG: Electrocardiograms
CT: Computed tomography
